# Impact of primary tumor clinicopathological factors on prognosis after hepatic resection for rectal liver metastases

**DOI:** 10.1002/ags3.12917

**Published:** 2025-01-24

**Authors:** Tomofumi Uotani, Takeshi Takamoto, Satoshi Nara, Daisuke Ban, Takahiro Mizui, Konosuke Moritani, Shunsuke Tsukamoto, Yukihide Kanemitsu, Tsutomu Fujii, Minoru Esaki

**Affiliations:** ^1^ Department of Hepatobiliary and Pancreatic Surgery National Cancer Center Hospital Tokyo Japan; ^2^ Department of Colorectal Surgery National Cancer Center Hospital Tokyo Japan; ^3^ Department of Surgery and Science, Faculty of Medicine, Academic Assembly University of Toyama Toyama Japan

**Keywords:** hepatectomy, neoplasm metastasis, peripheral nerves, rectal neoplasms, recurrence

## Abstract

**Background:**

Treatment of liver metastases from rectal cancer has been considered based on research data on liver metastases from colorectal cancer. This study aimed to clarify the impact of clinicopathological factors of the primary lesion, including rectal cancer‐specific factors such as lateral lymph node metastasis, on the prognosis after liver resection.

**Methods:**

This was a single‐center retrospective study of patients undergoing curative surgical treatment for resectable liver metastases from rectal cancer from January 2010 to June 2023. Prognostic factors were investigated using univariable and multivariable analyses.

**Results:**

The cohort consisted of 113 males and 44 females, with a median age of 60 years. Lateral lymph node dissection was performed in 48 patients, of which 11 had positive nodes. Multivariable analysis revealed lateral lymph node metastasis (HR 5.86; 95% CI 2.40–14.2; *p* = 0.0001) and perineural invasion (HR 2.83; 95% CI 1.36–5.88; *p* = 0.005) as independent prognostic factors. After curative hepatectomy, 73.3% of patients with these two factors showed early recurrence within 6 months, requiring nonsurgical treatment.

**Conclusions:**

Lateral lymph node metastasis and perineural invasion of the primary tumor were prognostic factors after resection of rectal liver metastases. Patients with these factors required nonsurgical treatment in the early postoperative period.

## Graphical abstract

This retrospective study evaluates the prognostic factors in patients undergoing curative hepatic resection for rectal cancer liver metastases. It identifies lateral lymph node metastasis and perineural invasion as significant prognostic factors. Moreover, patients with these factors required nonsurgical treatment in the early postoperative period.

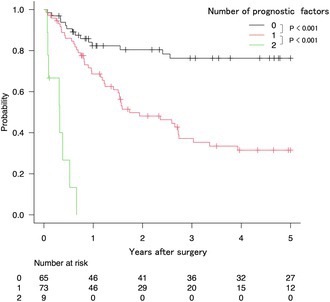

## 1 INTRODUCTION

Colorectal cancer is the third most common cancer and the second leading cause of cancer‐related deaths worldwide, and rectal cancer accounts for approximately 40% of them.^1^ Despite developments in the treatment of colorectal cancer, half of patients experience colorectal liver metastases during their disease.^2^, ^3^, ^4^ In rectal cancer, the incidence of synchronous liver metastases is reported as ranging from 14% to 18.4%.^5^, ^6^ The management of rectal liver metastases (RLM) is not distinct from colon liver metastases, and operability is judged based on the same criteria, such as nodule size and number, non‐tumoral liver condition, and future remnant liver volume.^7^, ^8^, ^9^ Therefore, the prognostic benefits of hepatic resection for colorectal liver metastases (CRLM) have been collectively reported.

Rectal cancer has complex anatomic characteristics, including lateral lymph nodes (LLN) and pelvic nerve plexus, and treatment strategies for advanced rectal cancer have varied by country and by facility, unlike colon cancer. Recently, advanced rectal cancer treatment strategies have become more complex with the establishment of approaches such as total neoadjuvant therapy and the “watch and wait” strategy. It remains uncertain whether the treatment strategy for colon liver metastases is applicable to cases of rectal cancer with liver metastases and how rectal cancer‐specific pathologic factors, such as lateral lymph node metastases, influence the prognosis.

In this study, we aimed to investigate the association between clinicopathological factors and prognosis following radical hepatic resection in patients with RLM and identify the patients requiring more multidisciplinary treatment.

## 2 MATERIALS AND METHODS

### 2.1 Patient selection

From a prospectively maintained database, we identified patients who underwent R0 or R1 hepatic resection for liver metastases from rectal cancer between January 2010 and June 2023. Patients with primary tumors at sites other than the rectum, those who underwent endoscopic resection of the primary site, and patients without primary tumor data were excluded from this study. Both synchronous and metachronous RLM were enrolled. The Institutional Review Board approved this study protocol (NCC no. 2018‐299, no. 2020‐275).

### 2.2 Treatment approach and perioperative management for liver metastases

The treatment strategy and perioperative management for CRLM were described previously.^10^ In brief, the resectability of the tumor and the surgical procedure were determined according to the decision‐making criteria based on tumor localization and liver function results from the preoperative indocyanine green retention test.^11^, ^12^ Preoperative portal vein embolization, staged hepatectomy, and hepatectomy with venous reconstruction were conducted for patients with a future liver remnant volume expected to be functionally inadequate. Patients did not routinely receive adjuvant chemotherapy after hepatectomy and were followed for 5 years with regular laboratory and imaging evaluations every 3–4 months. Routine neoadjuvant chemotherapy (NAC) for resectable CRLM was not performed, while the present study included patients who had resectable CRLM but received NAC at a previous hospital before consulting our institutions. The duration and regimens of NAC depended on prior physician decisions.

### 2.3 Treatment approach and perioperative management for rectal cancer

All patients received preoperative evaluation with endoscopy, ultrasonography, computed tomography (CT), and magnetic resonance imaging. Based on preoperative images and intraoperative findings, patients diagnosed as cT1 or T2 stage, and without evidence of LLN involvement, underwent standard total mesorectal excision (TME). Patients classified as cT3 or T4 stage with tumors located at or below the peritoneal reflection or those with suspected positive LLN, underwent LLN dissection in addition to TME. Neoadjuvant therapy was not performed, except for those with high‐risk factors of local recurrence, such as positive clinical circumferential resection margin or involving adjacent organs.

### 2.4 Statistical analyses

Continuous variables were compared using the Mann–Whitney *U* test or the Kruskal–Wallis test, while categorical variables were compared using the chi‐squared test. Survival curves were generated using the Kaplan–Meier method and compared using the log‐rank test. Differences in survival between groups were evaluated using Cox proportional hazards model analyses. Factors to be included in the multivariable analysis were selected based on a *p* value <0.10 in the univariable analysis or clinical relevancy. Hazard ratios (HRs) and 95% confidence intervals (CIs) were calculated for each factor. All statistical tests were two‐sided, and *p* < 0.05 was considered statistically significant. Statistical analyses were conducted with EZR (Saitama Medical Center, Jichi Medical University, Saitama, Japan).^13^

## 3 RESULTS

### 3.1 Patient characteristics

From January 2010 to June 2023, 157 patients were included in this study. The clinicopathological characteristics of the patients are summarized in Table 1. There were 113 males and 44 females with a median age of 60 (range 27–87) years. Seventy‐five patients (47.8%) had synchronous liver metastases and 82 patients (52.2%) had metachronous liver metastases. Twenty‐four of 157 patients (15.3%) received preoperative treatment prior to the resection of the primary tumor, including 16 patients who underwent chemotherapy and eight patients who received chemoradiotherapy. Before liver resection, CEA and CA19‐9 were elevated in 93 (63.3%) and 59 (39.9%) patients, respectively. The median size and number of liver metastases were 25 mm (range 3–105) and 2 (range 1–18). Eighteen patients (11.6%) had synchronous pulmonary metastases, and 35 patients (22.3%) underwent neoadjuvant chemotherapy before liver resection. Ten patients (28.6%) had technically unresectable liver metastases, and after chemotherapy, tumor shrinkage was achieved and radical hepatic resection was performed. Adjuvant chemotherapy following liver resection was administered to 29 of 157 patients (18.5%).

**TABLE 1:** Patient demographics and pathological characteristics.

Variable	*n* = 157
Age (years)	60 (27–87)
Sex, female	44 (28.0%)
Timing of liver metastasis, synchronous	75 (47.8%)
Liver metastasis size (mm)	25 (3–105)
Number of metastases	2 (1–18)
CEA (ng/mL)^a^	7.4 (0–2620)
CA19‐9 (U/mL)^a^	27.5 (1–4026)
Synchronous pulmonary metastasis	18 (11.6%)
Neoadjuvant chemotherapy	35 (22.3%)
Major hepatectomy^b^	20 (12.7%)
Postoperative complications^c^	18 (11.7%)
Adjuvant chemotherapy^d^	29 (18.5%)

Abbreviations: CEA, carcinoembryonic antigen; CA‐19, carbohydrate antigen 19–9.

^a^ Pre‐hepatectomy.

^b^ A major hepatectomy is defined as an anatomical liver resection consisting of three Couinaud's segments or more.

^c^ Clavien‐Dindo classification ≥ Grade 2.

^d^ Adjuvant chemotherapy following liver resection.

### 3.2 Primary tumor pathological characteristics

As shown in Table 2, there were 58 rectosigmoid (36.9%), 47 upper‐rectum (29.9%), and 52 lower‐rectum (33.1%) cancer cases. LLN dissection was performed in 48 patients (31%), of whom 11 patients (22.4%) had positive lymph nodes. Among the patients who did not undergo LLN dissection, four patients experienced LLN recurrence. Combining the 11 patients with positive lymph nodes and the four patients with LLN recurrence, a total of 15 patients were categorized into the potential LLN metastasis group. For the pathological T stage, three patients were T1, 11 patients were T2, 122 patients were T3, and 21 patients were T4. Primary site histologic grade was well or moderately differentiated in most patients (97.4%). About half of the patients (51.7%) had perineural invasion, and most patients (90.9%) had vascular invasion. RAS mutation status was not investigated or was unknown in approximately half of the patients.

**TABLE 2:** Primary tumor pathological characteristics.

Variable	*n* = 157
Location	
Rectosigmoid	58 (36.9%)
Upper rectum	47 (29.9%)
Lower rectum	52 (33.2%)
Tumor size (mm)	50 (12–140)
pT stage	
T1b	3 (1.9%)
T2	11 (7.0%)
T3	122 (77.7%)
T4a	14 (8.9%)
T4b	7 (4.5%)
pN status, > pN1	86 (55.1%)
Number of lymph nodes examined	23.5 (2–85)
Extra Deposits, positive	38 (27.9%)
Lateral lymph node dissection, performed	48 (31.0%)
Lateral lymph node metastasis, positive	11 (22.4%)
Potential lateral lymph node metastasis, positive^a^	15 (9.5%)
Histological grade	
Well	59 (37.8%)
Moderately	93 (59.6%)
Poorly	4 (2.6%)
Lymphatic invasion	56 (36.4%)
Vascular invasion	140 (90.9%)
Perineural invasion	76 (51.7%)
Budding grade	
1	72 (57.6%)
2	24 (19.2%)
3	29 (23.2%)
RAS status	
Wild	51 (60%)
Mutant	34 (40%)

Abbreviations: Extra Deposits, Extramural cancer deposits without lymph node structure; pN stage, pathological lymph node stage; pT stage, pathological tumor stage.

^a^ Eleven patients had pathological positive lymph nodes, and four patients experienced lateral lymph node recurrence.

### 3.3 Prognostic factor analysis

Table 3 shows the results of univariable and multivariable analysis. In univariable analysis, 4 or more liver metastases, synchronous liver metastases, primary T and N factors, LLN metastases, histological grade of the primary site, lymphatic invasion, perineural invasion, and chemotherapy before hepatectomy were associated with prognosis (*p* < 0.1). Multivariable analysis identified LLN metastasis (HR 5.86; 95% CI 2.40–14.2; *p* = 0.0001) and perineural invasion (HR 2.83; 95% CI 1.36–5.88; *p* = 0.005) as independent prognostic factors.

**TABLE 3:** Variables associated with overall survival according to the Cox proportional hazards regression model.

Variable	*n*	Univariable analysis		Multivariable analysis	
		HR (95% CI)	*P* value	HR (95% CI)	*P* value
Age (years)					
≤60	82	Reference			
>60	75	1.33 (0.78–2.26)	0.291		
Sex					
Female	44	Reference			
Male	113	1.50 (0.77–2.90)	0.229		
Liver metastasis variable					
Liver metastasis size (cm)					
≤3	97	Reference			
>3	60	1.05 (0.61–1.80)	0.857		
Number of metastases					
≤3	118	Reference		Reference	
>3	39	2.22 (1.27–3.89)	**0.005**	1.81 (0.99–3.31)	0.054
Timing of liver metastasis					
Synchronous	75	Reference		Reference	
Metachronous	82	0.45 (0.26–0.79)	**0.004**	0.74 (0.37–1.48)	0.400
Synchronous pulmonary metastasis					
Absent	137	Reference			
Present	18	1.34 (0.57–3.14)	0.494		
CEA (ng/mL)					
≤5	54	Reference			
>5	93	1.24 (0.70–2.20)	0.459		
CA19‐9 (U/mL)					
≤37	89	Reference			
>37	59	1.53 (0.90–2.60)	0.116		
Neoadjuvant chemotherapy					
Yes	35	Reference		Reference	
No	122	0.55 (0.29–1.04)	**0.067**	0.89 (0.38–2.05)	0.794
Major hepatectomy^a^					
No	137	Reference			
Yes	20	1.10 (0.49–2.44)	0.805		
Complications (Clavien‐Dindo classification)					
< Grade 2	136	Reference			
≥ Grade 2	18	1.19 (0.51–2.80)	0.675		
Primary site variable					
Location					
Rectosigmoid/Upper rectum	105	Reference			
Lower rectum	52	0.77 (0.43–1.38)	0.388		
pT stage					
T1‐2	14	Reference		Reference	
T3	122	3.67 (0.88–15.2)	**0.072**	1.98 (0.44–8.85)	0.369
T4	21	4.11 (0.90–18.8)	**0.068**	2.76 (0.53–14.2)	0.224
pN stage					
0	70	Reference		Reference	
≥1	86	1.63 (0.91–2.92)	**0.098**	0.63 (0.30–1.33)	0.231
Potential lateral lymph node metastasis					
Absent	142	Reference		Reference	
Present	15	4.27 (2.11–8.65)	**<0.001**	5.86 (2.40–14.2)	**< 0.001**
Primary site histological grade					
Well	59	Reference		Reference	
Moderate	93	1.57 (0.85–2.91)	0.146	1.05 (0.57–1.95)	0.308
Poorly	4	5.27 (1.50–18.4)	**0.009**	2.66 (0.99–7.13)	0.051
Lymphatic invasion					
Absent	98	Reference		Reference	
Present	56	2.43 (1.43–4.14)	**0.001**	1.46 (0.78–2.76)	0.233
Vascular invasion					
Absent	14	Reference			
Present	140	1.12 (0.40–3.10)	0.828		
Perineural invasion					
Absent	71	Reference		Reference	
Present	76	3.05 (1.65–5.64)	**<0.001**	2.83 (1.36–5.88)	**0.005**
Budding grade					
Grade 1	72	Reference			
Grade 2	24	0.77 (0.33–1.80)	0.553		
Grade 3	29	1.15 (0.58–2.26)	0.682		

*Note*: *p*‐values below 0.1 in the univariate analysis (Table 3) are highlighted in bold. For the multivariate analysis, *p*‐values of 0.05 or less are also presented in bold to indicate statistical significance.Abbreviations: CA‐19, carbohydrate antigen 19–9; CEA, carcinoembryonic antigen; CI, confidence interval; HR, hazard ratio; pN stage, pathological lymph node stage; pT stage, pathological tumor stage.

^a^ A major hepatectomy is defined as an anatomical liver resection consisting of three Couinaud's segments or more.

### 3.4 Prognostic impact of lateral lymph node metastases and perineural invasion

**FIGURE 1:**
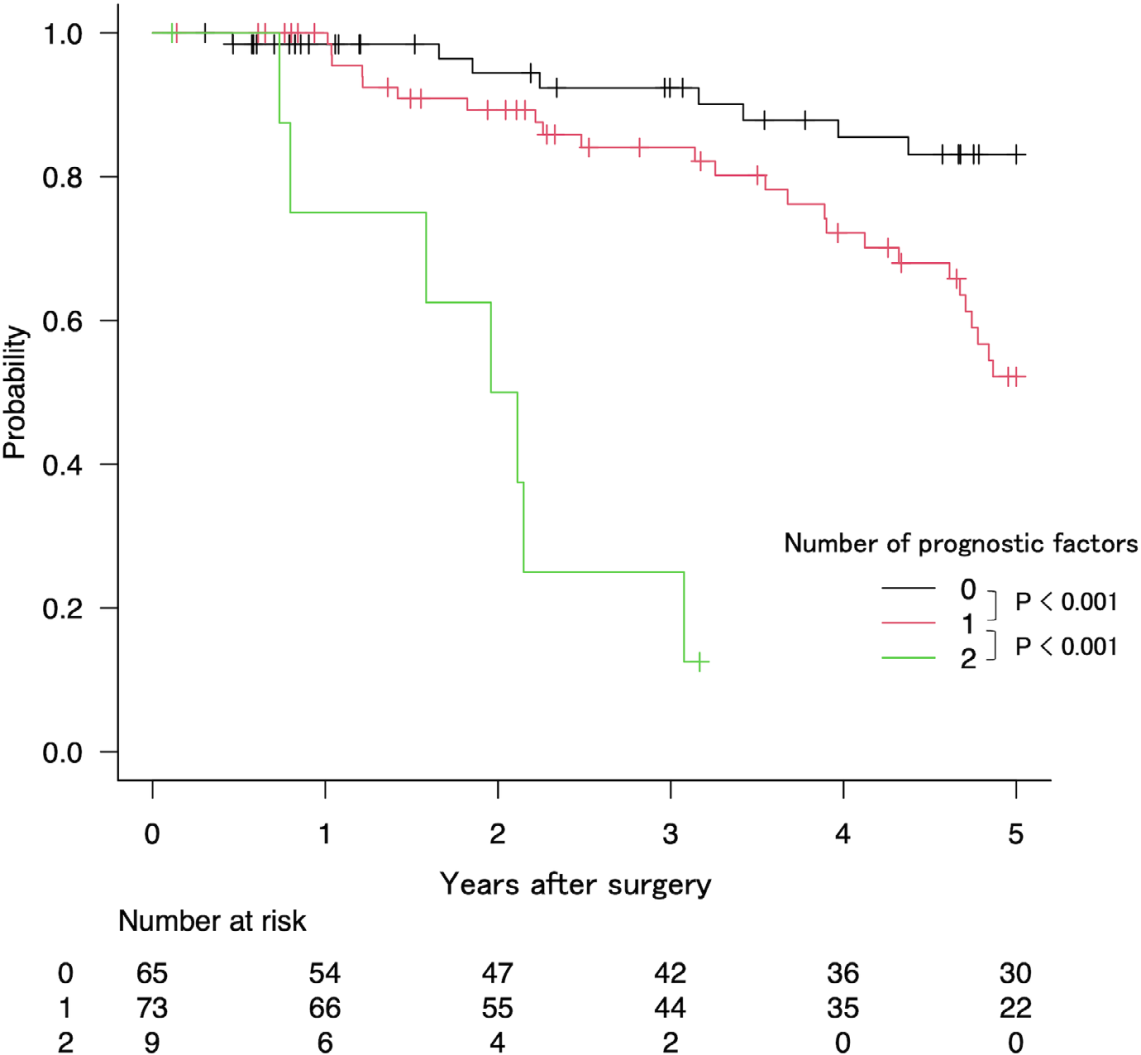
Kaplan–Meier plots of overall survival stratified according to the number of applicable prognostic factors: Lateral lymph node metastases and perineural invasion.

**FIGURE 2:**
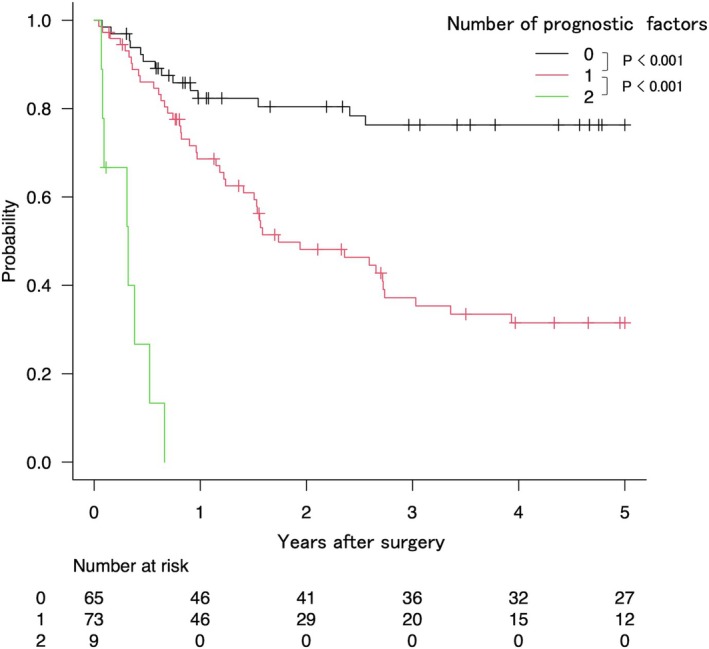
Kaplan–Meier plots of time to surgical failure stratified according to the number of applicable prognostic factors: Lateral lymph node metastases and perineural invasion.

The overall 5‐year overall survival (OS) and 5‐year recurrence‐free survival (RFS) rates were 62.3% (95% CI, 52.4–70.6) and 30.3% (95% CI, 22.7–38.1), respectively. Postoperative chemotherapy was administered to 29 patients (18.5%) following liver resection. Among these patients, the 5‐year OS and RFS rates were 26.0% (95% CI, 11.7–43.0) and 31.1% (95% CI, 22.6–39.9), respectively. In contrast, for patients who did not receive postoperative chemotherapy, the 5‐year OS and RFS rates were 46.9% (95% CI, 26.2–65.2) and 66.2% (95% CI, 55.0–75.2), respectively. However, the differences in OS and RFS between the groups were not statistically significant (OS: *p* = 0.132; RFS: *p* = 0.087). To further investigate prognostic factors, including LLN metastasis and perineural invasion, a total of 147 patients were analyzed after excluding cases with incomplete data. The median follow‐up time was 46.7 months. Figures 1 and 2 show the results of a Kaplan–Meier analysis for overall survival (OS) and time to surgical failure (TSF) after surgery stratified according to the number of prognostic factors in each case. Both the OS (Figure 1) and TSF (Figure 2) exhibited significant trends indicating worse outcomes as the number of prognostic factors increase (*p* < 0.001).

After curative hepatectomy, 73.3% of patients who had these two factors showed early recurrence within 6 months, requiring nonsurgical treatment. Recurrence patterns stratified by the number of risk factors are summarized in Table 4. Notably, multiorgan recurrence was observed more frequently with an increasing number of risk factors.

**TABLE 4:** The pattern of recurrence stratified by the number of prognostic factors.

Number of prognostic factors	0 (*n* = 31)	1 (*n* = 57)	2 (*n* = 8)
Single organ recurrence	25 (80.6%)	39 (68.4%)	4 (50.0%)
Liver metastasis	14 (56.0%)	18 (46.2%)	0 (0.0%)
Pulmonary metastasis	7 (28.0%)	15 (38.5%)	0 (0.0%)
Local recurrence	2 (8.0%)	1 (2.6%)	0 (0.0%)
Lymph node metastasis	2 (8.0%)	3 (7.7%)	3 (75.0%)
Other^a^	0 (0.0%)	2 (5.1%)	1 (25.0%)
Multiorgan recurrence	6 (19.4%)	18 (31.6%)	4 (50.0%)
Liver and pulmonary metastasis	3 (50.0%)	6 (33.3%)	1 (25.0%)
Other^b^	3 (50.0%)	12 (66.7%)	3 (75.0%)

^a^ Born and peritoneal.

^b^ The involvement of two or more sites, including liver and pulmonary, lymph nodes, adrenal glands, peritoneum, brain, renal, and local recurrence.

## 4 DISCUSSION

In this study, we investigated prognostic determinants following liver metastasis resection in rectal cancer cases. Our findings indicate that the primary tumor factors, specifically LLN metastases and perineural invasion, were associated with a worse prognosis than the liver metastasis factors. Patients with these two factors demonstrated poor prognosis and a high rate of early disease recurrence beyond surgical retreatment.

Our results indicated that the characteristics of the primary site may be equally or even more effective than those of liver metastasis in predicting the prognosis of rectal cancer liver metastases. Several prognostic factors after curative resection of CRLM have been documented. These encompass factors associated with liver tumors, such as the maximum diameter and the number of liver metastases, the grade of differentiation, and the surgical margin status, in addition to serum CEA levels and the disease‐free interval from primary resection. Pathologic factors of the primary tumor, such as advanced T stage, nodal status, and tumor location (the right‐sided or left‐sided colons as the primary lesion), have also been linked to an unfavorable prognosis.^14^, ^15^, ^16^, ^17^ In the present study, focusing on rectal cancer, only the primary tumor factors of positive LLN metastases and perineural invasion were independent prognostic factors. In contrast, the factors of liver metastasis did not remain as independent prognostic factors after multivariate analysis. These two primary site factors have been reported to be associated with postoperative local recurrence and prognosis in resectable advanced rectal cancer.^18^, ^19^, ^20^, ^21^, ^22^

Our findings in TSF can contribute to making a treatment strategy for rectal cancer with resectable liver metastases. The assessment of postoperative outcomes in liver metastases often incorporates the concept of TSF, as suggested in previous literature.^23^ This concept is advocated because of its superior prognostic accuracy compared to assessing the time to recurrence. In our study, we noted a recurrence rate of 73.3% within 6 months post‐surgery, escalating to 100% within a year among patients exhibiting two adverse prognostic indicators. This finding highlights a notable clinical challenge in the context of radical hepatectomy for this patient cohort. Despite the technical feasibility of such surgical interventions, our data indicate a predisposition to early postoperative recurrence. Therefore, given the substantial likelihood of early recurrence, the indication for surgery in these patients demands careful evaluation, and intensive postoperative treatments such as adjuvant chemotherapy should be considered.

This study has several limitations. First, its retrospective nature and relatively small number of patients may introduce potential biases. Second, genetic profiles recognized as prognostic factors after curative resection of CRLM are not evaluated. Although it is controversial whether RAS mutation status contributes to the prognosis after hepatic resection of CRLM, RAS status information was unavailable for all cases in the present study.

In conclusion, in RLM cases with lateral lymph node metastases and perineural invasion, even if resectable, the low likelihood of cure with surgery alone suggests the need to consider systemic therapy in the treatment strategy.

## AUTHOR CONTRIBUTIONS

**Tomofumi Uotani:** Conceptualization; data curation; formal analysis; investigation; writing – original draft. **Takeshi Takamoto:** Conceptualization; data curation; formal analysis; investigation; methodology; writing – original draft; writing – review and editing. **Satoshi Nara:** Writing – review and editing. **Daisuke Ban:** Writing – review and editing. **Takahiro Mizui:** Writing – review and editing. **Konosuke Moritani:** Writing – review and editing. **Shunsuke Tsukamoto:** Writing – review and editing. **Yukihide Kanemitsu:** Writing – review and editing. **Tsutomu Fujii:** Writing – review and editing. **Minoru Esaki:** Writing – review and editing.

## FUNDING INFORMATION

The authors have nothing to report.

## CONFLICT OF INTEREST STATEMENT

Tsutomu Fujii is an editorial board member of the Annals of Gastroenterological Surgery.

## ETHICS STATEMENT

Approval of the research protocol: The Institutional Review Board approved this study protocol (NCC no. 2018‐299, no. 2020‐275).

Informed Consent: N/a.

Registry and the Registration No. of the study/trial: N/a.

Animal Studies: N/a.

## References

[1] Global Cancer Observatory . International Agency for Research on Cancer. World Health Organization. Accessed on November 19, 2023. https://gco.iarc.fr/.

[2] Adam R , de Gramont A , Figueras J , Kokudo N , Kunstlinger F , Loyer E , et al. Managing synchronous liver metastases from colorectal cancer: a multidisciplinary international consensus. Cancer Treat Rev. 2015;41:729–741. 26417845 10.1016/j.ctrv.2015.06.006

[3] Ali SM , Pawlik TM , Rodriguez‐Bigas MA , Monson JRT , Chang GJ , Larson DW . Timing of surgical resection for curative colorectal cancer with liver metastasis. Ann Surg Oncol. 2018;25:32–37. 28224365 10.1245/s10434-016-5745-7

[4] Tsilimigras DI , Brodt P , Clavien PA , Muschel RJ , D'Angelica MI , Endo I , et al. Liver metastases. Nat Rev Dis Primers. 2021;7(1):27. 33859205 10.1038/s41572-021-00261-6

[5] Wade TP , Virgo KS , Li MJ , Callander PW , Longo WE , Johnson FE . Outcomes after detection of metastatic carcinoma of the colon and rectum in a national hospital system. J Am Coll Surg. 1996;182:353–361. 8605559

[6] Weber JC , Bachellier P , Oussoultzoglou E , Jaeck D . Simultaneous resection of colorectal primary tumour and synchronous liver metastases. Br J Surg. 2003;90:956–962. 12905548 10.1002/bjs.4132

[7] Schmoll HJ , Van Cutsem E , Stein A , Valentini V , Glimelius B , Haustermans K , et al. ESMO consensus guidelines for management of patients with colon and rectal cancer. A personalized approach to clinical decision making. Ann Oncol. 2012;23:2479–2516. 23012255 10.1093/annonc/mds236

[8] Hashiguchi Y , Muro K , Saito Y , Ito Y , Ajioka Y , Hamaguchi T , et al. Japanese Society for Cancer of the Colon and Rectum (JSCCR) guidelines 2019 for the treatment of colorectal cancer. Int J Clin Oncol. 2019;25(1):1–42. 31203527 10.1007/s10147-019-01485-z PMC6946738

[9] National Comprehensive Cancer Network . Rectal cancer (Version 6.2023). Accessed on November 19, 2023. https://www.nccn.org/professionals/physician_gls/pdf/rectal.pdf

[10] Sakamoto Y , Fujita S , Akasu T , Nara S , Esaki M , Shimada K , et al. Is surgical resection justified for stage IV colorectal cancer patients having bilobar hepatic metastases?—an analysis of survival of 77 patients undergoing hepatectomy. J Surg Oncol. 2010;102:784–788. 20872814 10.1002/jso.21721

[11] Imamura H , Seyama Y , Kokudo N , Maema A , Sugawara Y , Sano K , et al. One thousand fifty‐six hepatectomies without mortality in 8 years. Arch Surg. 2003;138:1198–1206. 14609867 10.1001/archsurg.138.11.1198

[12] Takamoto T , Sano K , Hashimoto T , Ichida A , Shimada K , Maruyama Y , et al. Practical contribution of virtual hepatectomy for colorectal liver metastases: a propensity‐matched analysis of clinical outcome. J Gastrointest Surg. 2018;22:2037–2044. 29980979 10.1007/s11605-018-3860-4

[13] Kanda Y . Investigation of the freely available easy‐to‐use software ‘EZR’ for medical statistics. Bone Marrow Transplant. 2013;48(3):452–458. 23208313 10.1038/bmt.2012.244 PMC3590441

[14] Fong Y , Fortner J , Sun RL , Brennan MF , Blumgart LH . Clinical score for predicting recurrence after hepatic resection for metastatic colorectal cancer: analysis of 1001 consecutive cases. Ann Surg. 1999;230:309. 10493478 10.1097/00000658-199909000-00004 PMC1420876

[15] Ribero D , Viganò L , Amisano M , Capussotti L . Prognostic factors after resection of colorectal liver metastases: from morphology to biology. Future Oncol. 2013;9:45–57. 23252563 10.2217/fon.12.159

[16] Sasaki K , Andreatos N , Margonis GA , He J , Weiss M , Johnston F , et al. The prognostic implications of primary colorectal tumor location on recurrence and overall survival in patients undergoing resection for colorectal liver metastasis. J Surg Oncol. 2016;114:803–809. 27792291 10.1002/jso.24425

[17] Jones RP , Brudvik KW , Franklin JM , Poston GJ . Precision surgery for colorectal liver metastases: opportunities and challenges of omics‐based decision making. Eur J Surg Oncol. 2017;43:875–883. 28302330 10.1016/j.ejso.2017.02.014

[18] Homma Y , Hamano T , Otsuki Y , Shimizu S , Kobayashi Y . The total number of lymph node metastases is a more significant risk factor for poor prognosis than positive lateral lymph node metastasis. Surg Today. 2015;45:168–174. 24831659 10.1007/s00595-014-0913-5

[19] Knijn N , Mogk SC , Teerenstra S , Simmer F , Nagtegaal ID . Perineural invasion is a strong prognostic factor in colorectal cancer: a systematic review. Am J Surg Pathol. 2016;40:103–112. 26426380 10.1097/PAS.0000000000000518

[20] Yagi R , Shimada Y , Kameyama H , Tajima Y , Okamura T , Sakata J , et al. Clinical significance of extramural tumor deposits in the lateral pelvic lymph node area in low rectal cancer: a retrospective study at two institutions. Ann Surg Oncol. 2016;23:552–558. 10.1245/s10434-016-5379-9 PMC5035319 27393567

[21] Alotaibi AM , Lee JL , Kim J , Lim SB , Yu CS , Kim TW , et al. Prognostic and oncologic significance of perineural invasion in sporadic colorectal cancer. Ann Surg Oncol. 2017;24:1626–1634. 28070726 10.1245/s10434-016-5748-4

[22] Zhou S , Jiang Y , Pei W , Liang J , Zhou Z . Prognostic significance of lateral pelvic lymph node dissection for middle‐low rectal cancer patients with lateral pelvic lymph node metastasis: a propensity score matching study. BMC Cancer. 2022;22:1–10. 35109810 10.1186/s12885-022-09254-4 PMC8812196

[23] Oba M , Hasegawa K , Matsuyama Y , Shindoh J , Mise Y , Aoki T , et al. Discrepancy between recurrence‐free survival and overall survival in patients with resectable colorectal liver metastases: a potential surrogate endpoint for time to surgical failure. Ann Surg Oncol. 2014;21:1817–1824. 24499828 10.1245/s10434-014-3504-1

